# Physiological and fitness differences between cytotypes vary with stress in a grassland perennial herb

**DOI:** 10.1371/journal.pone.0188795

**Published:** 2017-11-30

**Authors:** Zuzana Pavlíková, Dana Holá, Blanka Vlasáková, Tomáš Procházka, Zuzana Münzbergová

**Affiliations:** 1 Department of Botany, Faculty of Science, Charles University, Prague, Czech Republic; 2 Department of Genetics and Microbiology, Faculty of Science, Charles University, Prague, Czech Republic; 3 Department of Population Ecology, Institute of Botany, Academy of Sciences of the Czech Republic, Průhonice, Czech Republic; National Cheng Kung University, TAIWAN

## Abstract

**Background and aims:**

Understanding the consequences of polyploidization is a major step towards assessing the importance of this mode of speciation. Most previous studies comparing different cytotypes, however, did so only within a single environment and considered only one group of traits. To take a step further, we need to explore multiple environments and a wide range of traits. The aim of this study was to assess response of diploid and autotetraploid individuals of *Knautia arvensis* (Dipsacaceae) to two stress conditions, shade or drought.

**Methods:**

We studied eleven photosynthetic, morphological and fitness parameters of the plants over three years in a common garden under ambient conditions and two types of stress.

**Key results:**

The results indicate strong differences in performance and physiology between cytotypes in ambient conditions. Interestingly, higher fitness in diploids contrasted with more efficient photosynthesis in tetraploids in ambient conditions. However, stress, especially drought, strongly reduced fitness and disrupted function of the photosystems in both cytotypes reducing the between cytotype differences. The results indicate that drought stress reduced function of the photosynthetic processes in both cytotypes but particularly in tetraploids, while fitness reduction was stronger in diploids.

**Conclusions:**

The photosynthesis related traits show higher plasticity in polyploids as theoretically expected, while the fitness related traits show higher plasticity in diploids especially in response to drought. This suggests that between cytotype comparisons need to consider multiple traits and multiple environments to understand the breath of possible responses of different cytotypes to stress. They also show that integrating results based on different traits is not straightforward and call for better mechanistic understanding of the relationships between species photosynthetic activity and fitness. Still, considering multiple environments and multiple species traits is crucial for understanding the drivers of niche differentiation between cytotypes in future studies.

## Introduction

Polyploidy is an important genomic change for all eukaryotes [1]. In plants, it is estimated that polyploidy occurs in 50–70% of angiosperms [2]. It has been suggested that polyploidy represents a major driver of plant evolution underlying wide range of speciation events [3–6]. However, more sets of chromosomes and genes often cause genome instabilities, chromosome imbalances, regulatory incompatibilities and reproductive failures [7]. Therefore, a new polyploid line must undergo rapid changes in genome structure and gene expression after polyploidization to successfully establish (e.g. [8, 9]).

New polyploid lineage may arise either due to allopolyploidization or autopolyploidization. Alloploidization includes both the polyploidization event and hybridization, while autopolyploidization only includes the polyploidization event [10]. Consequences of both types of polyploidization have important evolutionary and ecological implications [11]. We, however, focus mostly on autopolyploidization as it allows exploring direct consequences of polyploidization without the necessity to consider the effects of hybridization. The studies on autopolyploids explored below include studies on established autopolyploids as well as de novo created synthetic autopolyploids and various cultivars.

Some of the most important consequences of autopolyploidization are higher genetic variability (diversification of duplicated genes), increased heterozygosity and increased allelic diversity and enzyme multiplicity compared to diploids as shown in a range of naturally occurring autopolyploids (e.g. [12–16]). Accordingly, polyploids should be able to generate a wide range of different responses to various abiotic and biotic stress factors, which could contribute to their evolutionary success and diversification [17].

Several recent studies explored differences in response of diploid and autopolyploid species pairs to various types of stress but the results are inconsistent. Specifically, autopolyploids were less sensitive to drought than diploids in most cases (e.g. [18] for natural and e.g. [19, 20] for in vitro-induced autopolyploids). In other studies, there were no differences (polyploid cultivars in [21], natural polyploids in [22]) or the pattern was reversed (polyploid cultivars [21], natural polyploids [23]). Similarly, diploids were less sensitive to shade than polyploid cultivars in Frydrych [24], while Sano [25] indicated the opposite. Petit and Thompson [26] did not find any differences between natural diploids and polyploids. This may suggest that the patterns are largely species specific. There is, however, no clear indication of differences between natural polyploids and polyploid cultivars.

The response of different cytotypes to stress is mediated by changes at wide range of traits occurring after polyploidization ranging from short-term changes in physiology and biochemistry to long-term changes in morphology and fitness. It was observed that autopolyploids have a higher number of chloroplasts and higher amount of chlorophyll and ribulose-1,5-bisphosphate carboxylase/oxygenase (Rubisco) enzyme [27] for a range of cultivated species) and they have bigger and fewer stomata (e.g., [28] for natural polyploids). Autopolyploid plants are also often larger and tend to have higher fitness as indicated in arrange of studies on natural polyploids [26, 29–32], but exceptions from this rule exist (e.g., [33, 34]).

Given the wide range of possible plant traits to be studied, it is possible that the variation in the results of different studies exploring stress response of different cytotypes may be also caused by use of different, and usually only few, plant traits in each study and the response is very trait specific. Specifically, it can be expected that physiological traits have much higher plasticity and more directly reflect processes within single cells and are thus more likely to show predictable responses across different environments and cytotypes. In contrast, long-term growth and fitness related traits are a result of an interplay between a wide range of different processes within a plant body and are thus more likely variable across different environments and cytotypes. If this is true when comparing different traits, however, remains to be tested.

Understanding how the physiological traits translate to growth and fitness related traits is crucial for our ability to predict future fates of different cytotypes. Multiple physiological, i.e. short-term, as well as long-term growth and fitness related plant traits and their mutual interplay should thus be considered to understand the specific mechanisms behind the response. Most of the existing studies exploring differences between cytotypes in response to stress are, however, focused on either physiology and morphology of plants [20, 35–40] or fitness traits [22, 41, 42]. Only a single study focused on both physiological and fitness traits in the same experiments [43]. It, [43], however, only focused on a single stress factor and a single physiological trait over only 6 weeks of plant life.

To study the effect of stress on performance of different cytotypes, we selected drought and shade as the stress factors. These two stress factors represent two major stresses affecting performance of species of semi natural grasslands [44] including our model species, *Knautia arvensis*. Drought and shade are also very important abiotic stress factors that strongly affect short term (physiology) as well as long-term (growth and fitness) performance of plants [45]. Drought stress usually causes a significant reduction in water potential and stomatal conductance for CO_2_ due to stomatal closure [46]. This has negative effect on photosynthetic rate and carboxylation efficiency (e.g., [20, 36, 37]) caused mostly by inhibition of secondary photosynthetic processes and ATP synthesis impairment [47, 48]. Other metabolic changes occur in chlorophyll and carotenoid content [35, 36] and in reactive oxygen metabolism [20]. All these physiological changes have many consequences for growth of the plants. Drought also causes a significant reduction in plant size as well as in the content of nitrogen in the leaves [49–51]. Exposure to shade also decreases relative growth rate of plants and their photosynthetic capability and diminishes reproductive potential by delaying initiation of flowering, decreasing flower and fruit yield and reducing plant biomass [42, 52].

The aim of this study was to compare diploid and autotetraploid individuals of *K*. *arvensis* under two stress conditions, shade or drought. *K*. *arvensis* was selected as a model as a typical representative of diploid-autotetraploid species with a contact zone in our study area, the Czech Republic, occurring in habitats with multiple stress factors [53]. We studied several morphological and physiological traits (photosynthetic parameters) and fitness of the plants over three years. Specifically, we wanted to answer the following questions: 1) Are there any between cytotype differences in response to stress?, 2) Can we distinguish any specific physiological mechanisms or traits, which are responsible for differences in stress response and which are related to ploidy level?

To explore the questions, we selected a range of traits related to species photosynthetic capacity including content of photosynthetic pigments, specific leaf mass, stomata size and several traits related to plant size and flowering to describe plant fitness. While other traits such as water use efficiency could also be important drivers of species response to drought, it has not been assessed in the current study. We hypothesize that polyploids generally have a wider capacity to respond to various environmental conditions, so we expect to observe less of a fitness reduction in the two stress conditions compared to diploids. The higher capacity of tetraploids to respond to stress could be thanks to the fact that tetraploids have a more flexible photosynthetic apparatus.

## Materials and methods

The study did not involve any protected species, so no permission was required. Also, the sampling sites were not protected and were freely accessible, so no permission was need to access the sites.

### Study species

*Knautia arvensis* (L.) Coult. s. str. (Dipsacaceae) is a perennial herb growing mainly in dry and mesophilous grasslands belonging to classes *Molinio-Arrhenatheretea* and *Festuco-Brometea*, but also in open woods and along roadsides [54]. The species is widely distributed in Europe and adjacent areas of Asia [54] and harbors two ploidy levels with more or less parapatric distribution and a contact zone running through Central Europe [53]. Plants have a sympodial shortened rootstock, simple or branched stems carrying violet, pink or pale yellow flower heads. The species is gynodioecious, having both hermaphroditic and female flowers [54].

### Study populations

**Fig 1:**
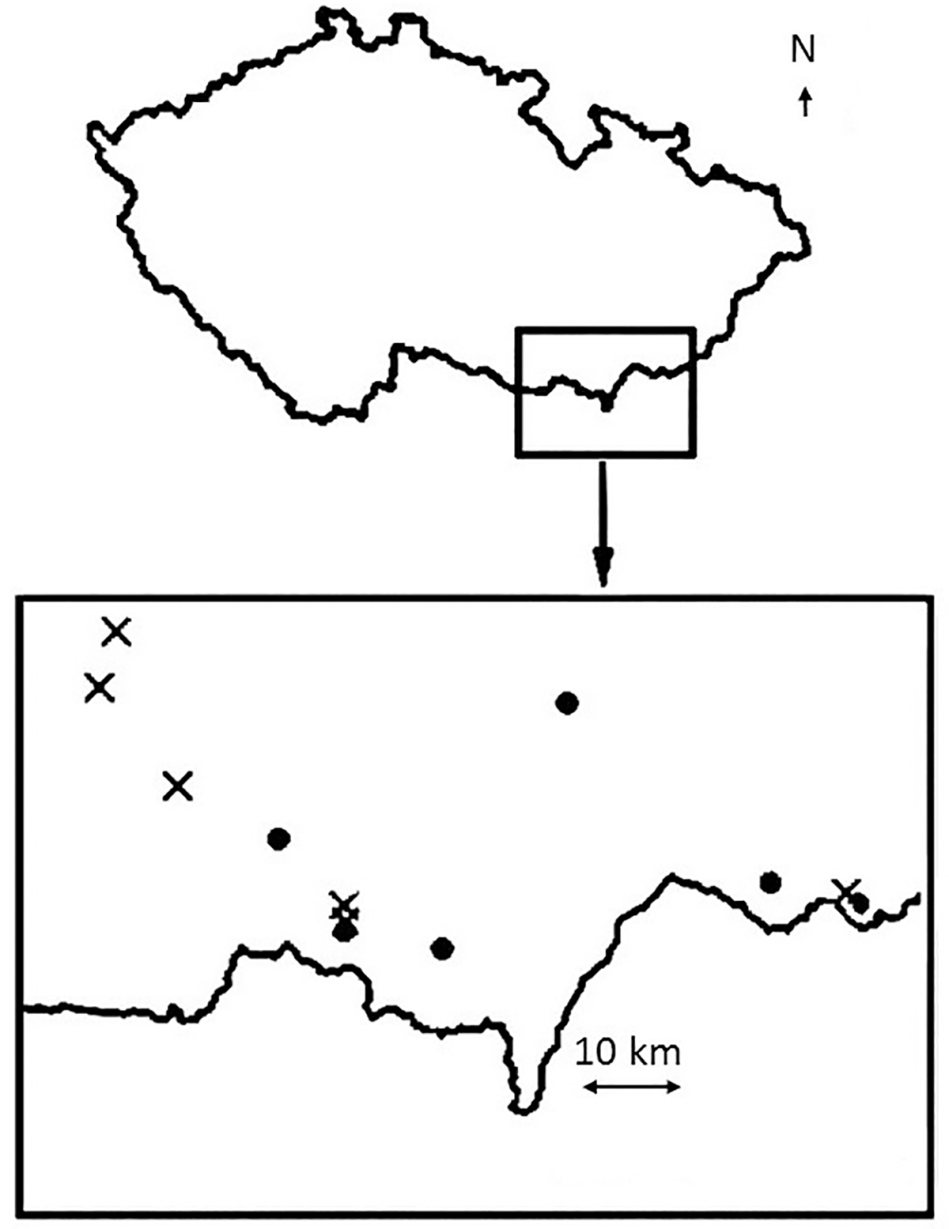
Map of the populations. Locations of the diploid (crosses) and tetraploid (circles) study populations in the Czech Republic, Europe.

For each cytotype, seeds were collected from several populations (6 populations for diploids and 5 populations for tetraploids, no mixed populations were available for collection) in 2009. All the populations were situated in open mesophylous grassland habitats. Populations were in the south-east of the Czech Republic, Europe (Fig 1). The information on the localities was obtained from [53]. The specific populations were selected as typical semi-natural grassland populations of the species, which were not mown at the time of seed collection, and we could thus collect sufficient number of ripe seeds for the experiment. Vegetation composition and Ellenberg indicator values derived from the vegetation composition [55] suggest that the sampled localities of tetraploids were drier and more shaded. The diploid localities were more variable ranging from drier to wetter and shaded to non-shaded conditions (our unpublished data). Ploidy level of all the experimental plants was estimated by the means of flow cytometry as described in [56]. All the diploid and tetraploid populations were used for the analysis of chlorophyll fluorescence, determination of photosynthetic pigment content and measurements of growth parameters. Only three diploid and three tetraploid populations were used for measurements of stomatal length for logistic reasons.

### Experimental set-up

From each population, we collected seeds from 10 maternal plants and kept seeds from each maternal plant separately. The maternal plants were selected to be at least 5 m apart. The collected seeds were sorted after collection and we selected maternal plants with at least 10 visually developed undamaged seeds available. The collected seeds were stored at 4°C in a refrigerator on wet filter paper in Petri dishes for three months (December 2009 –February 2010). In March 2010, seeds were sown into 8 × 8 × 8 cm pots filled with a commercial sowing substrate mixed with sand (2:3). Seeds from one maternal plant were placed into one pot. In total, seeds from 25 diploid and 25 tetraploid maternal plants were sown. These maternal plants originated from 6 different populations for diploids and 5 different populations for tetraploids with 2 to 5 maternal plants per population. The pots were kept in a heated greenhouse (18°C during the day, 10°C in the night). When the seedlings were large enough for manipulation (after approximately 1 month), three plants per sowing pot were individually transplanted to 16 × 16 × 16 cm pots filled with mixture of garden soil and sand (2:1). The common garden soil comprised of compost from the experimental garden containing approximately 0.135% of nitrogen, 1.35% of carbon and 46.5 mg of phosphorus in 1000 g of soil. We tried to choose equally sized seedlings. As at least three seeds successfully germinated from each maternal plant, we had 3 pots from 25 maternal plants (half-sib families) for each cytotype for the experiment. The pots with plants were placed in the experimental garden of the Institute of Botany, Czech Academy of Science, Průhonice, Czech Republic in May 2010, regularly watered and the plants were let to establish and grow for the whole field season of 2010.

In spring 2011, the diploid and tetraploid individuals were subjected to three different treatments–control, drought and shade. The three individuals originating from each maternal plant were divided among the three treatments. Thus, the maternal families were not replicated within treatments and the effect of maternal family could not be tested (but was used as a random factor, see below). However, this design ensured that we had the same maternal plants represented in all the treatments and the differences among treatments were not caused by different maternal plants used. The number of replicates per treatment thus corresponded to the number of maternal plants, i.e. 25 replicates for each cytotype and treatment, i.e. 150 pots in total.

Control plants were not shaded at all and were watered daily. Shaded plants were covered by a dense dark green mesh (65% light reduction without changes in red-far red ratio, [57] and were watered daily. Plants subjected to drought treatment were watered only when at least a single plant in the drought treatment started wilting (these plants received only about 5% of water compared to the watered and shaded plants, not counting the natural rainfall obtained by all the plants). While we did not monitor the conditions in our experiment in detail, another experiment with the same treatments in the same garden (Florianová and Münzbergová, submitted), indicated that shading reduced temperature by about 0.5°C and increased moisture by 7%. Drought stress reduced water availability by 32% and did not affect temperature.

Both drought and shade stress were applied to the plants from spring 2011 throughout the whole experiment until its end in 2014. In 2011, the plants did not flower. In 2012 and 2013, we recorded the number of flower heads and the number of flowering stalks produced by each individual over the whole growing season. In 2013, we also measured the height of the plants and the effective quantum yield of photosystem II (PSII) photochemistry in the light-adapted leaves (Qy). The polyphasic rise of chlorophyll fluorescence transient (OJIP) in the dark-adapted leaves and the content of photosynthetic pigments were measured on the same plants in 2014 (for methods of measurements, see below).

### Chlorophyll fluorescence analysis

Photosynthetic activity was measured in 42 diploid and 51 tetraploid plants with at least 12 individuals per cytotype and treatment. In all cases, the individuals originated from at least 3 populations and the same maternal plants were used from each treatment. Photosynthetic activity was not measured in all the plants as these measurements are very time consuming and need to be done only in a limited part of the day and comparable weather conditions. The imbalance in the final sample arose due to technical problems with our FluorPen device. As measuring photosynthesis is complicated, we have chosen an analysis of the rapid part of chlorophyll fluorescence induction kinetics (OJIP analysis) for our experiments, together with measurements of the effective quantum yield of photosystem (PS) II photochemistry (Qy). These non-destructive methods are the fastest ones that can be used for assessment of the photosynthetic processes, at the same time informing well on plant stress response. As PSII (together with other components of photosynthetic electron transport chain) is among the first sites where stress-induced damage can occur in plants, the characterization of the efficiency of the primary photosynthetic processes is a good way to assess plant response to any stress factor. Indeed, Qy is the basic parameter used for description of the stress level of an individual plant and one of OJIP parameters called PI_ABS_ (see below) is often recommended as a suitable marker for the determination of plant response to various stressors (e.g., [58–61]).

The OJIP analysis is based on the theory of energy flow in the photosynthetic electron-transport chain [62] and yields many numerical (JIP test) parameters that are calculated from chlorophyll fluorescence values determined at selected time points along the fluorescence induction kinetics curve (described, e.g., by [63]). The calculations and the biological meanings of the individual parameters of the JIP test are given in S1 Table. It can be also extended into a graphical analysis using various normalizations of the whole fluorescence induction kinetics curves (relative variable fluorescences W; [64]. Combination of both types of analyses then inform in detail on the state of individual parts of photosystem PS (II) complex as well as other components of photosynthetic electron-transport chain.

Qy and OJIP analysis were performed using the portable fluorometer FluorPen 100max (Photon Systems Instruments, Czech Republic). The measurements took place during the morning (between 9:00 and 11:00 AM). Each plant was represented by two randomly selected fully developed leaves and the chlorophyll fluorescence was measured on their adaxial surface in the middle part of the leaf blade two times for each leaf; these values were then averaged. The intensity of the saturating pulse (blue light, 455 nm) was 3,000 μmol m^-2^ s^-1^. All fluorescence transients were recorded with a time scan from 10 μs to 2 ms (the data acquisition rate was 1 reading per 10 μs for the first 600 μs, 1 reading per 100 μs till t = 14 ms, 1 reading per 1 ms till t = 90 ms and 1 reading per 10 ms for the rest of the recording period). The values of fluorescence recorded at 40 μs (F_0_, the initial fluorescence intensity), 300 μs (F_K_, the fluorescence intensity at the K-step), 2 ms (F_J_, the fluorescence intensity at the J-step), 30 ms (F_I_, the fluorescence intensity at the I-step), and F_M_ ≈ F_P_ (the maximum fluorescence intensity) were used for the calculations of various JIP test parameters. Qy parameter was calculated as (F_M_-F_0_)/F_M_ from the fluorescence values measured in light-adapted (i.e., not pre-darkened) leaves, all other parameters were calculated for dark-adapted (20 min in complete darkness) plants. Two performance indices (PI_ABS_ representing the energy conservation from photons absorbed by the photosystem II light-harvesting antenna to the reduction of Q_B_ plastoquinone electron acceptor of photosystem II and PI_TOTAL_ representing energy conservation from photons absorbed by the photosystem II antenna until the reduction of electron acceptors at the end of whole linear electron-transport chain) were selected as the best OJIP parameters characterizing the performance of primary photosynthetic processes.

The relative variable fluorescences W were calculated using the following expressions: W_OI_ = (F_t_-F_0_)/(F_I_-F_0_), W_OJ_ = (F_t_-F_0_)/(F_J_-F_0_), W_OK_ = (F_t_-F_0_)/(F_K_-F_0_) and W_IP_ = (F_t_-F_I_)/(F_P_-F_I_), where F_t_ represents the fluorescence intensity measured at any individual time during the recording period. The other abbreviations are explained above. The relative positions of the individual W_OI_, resp. W_IP_ curves were used for the comparisons of the rate of reduction of electron acceptors at the end of whole photosynthetic electron-transport chain and the size of the available pool of these acceptors, respectively (if the W_IP_ curve is positioned more to the right side of the graph, it means slower reduction of the end electron acceptors, whereas the lower position of the W_OI_ curve means smaller size of the pool of these electron acceptors [64]. W_OJ_ and W_OK_ were calculated in order to enable further calculations of the difference kinetics ΔW_OJ_ = (W_OJ STRESS_ -W_OJ CONTROL_) and ΔW_OK_ = (W_OK STRESS_-W_OK CONTROL_) to better reveal the K- and the L-bands. We used these difference kinetics to compare the plants exposed to shade or drought treatments with the control plants. K- band informs about a possible inactivation of the oxygen-evolving complex of photosystem II or/and about an increase in the size of a functional photosystem II antennae. L-band offers an information about excitonic connectivity among individual photosystem II units. If these two difference kinetics curves assume positive values when comparing stress with control, it means that the stressed plants have poorer excitonic connectivity than the control plants (ΔW_OK_) or that their oxygen-evolving complex is more inactivated/their antennae size has increased (ΔW_OJ_). The negative values would mean that stressed plants would perform better in these two aspects of primary photochemistry compared with the control ones [64].

### Content of chlorophyll and carotenoids

To estimate content of chlorophyll and carotenoids, four leaf discs (diameter 5 mm) were cut from the middle part of the leaf blade (3 leaves per plant, 7 plants per treatment and ploidy level originating from 3 different populations, the same maternal plants used in each treatment) and placed in test tubes containing 5 cm^3^ of N,N-dimethylformamide and stored at 4°C in the dark. After 7 days (during this period the test tubes were vortexed several times to allow for better extraction of pigments) the contents of chlorophylls (Chl) *a* and *b* and the content of total carotenoids (Car) were determined spectrophotometrically [65]. Additionally, four leaf discs were cut from the same leaves, oven-dried at 80ºC for 72 h and used for the determination of specific leaf mass (SLM, dry mass per leaf area unit). The mean values calculated for each individual plant from the respective three leaves were used for the statistical analyses.

### Stomatal length

Stomatal length was measured on 15 diploid and 15 tetraploid plants (5 maternal plants from 3 different populations per cytotype, control treatment only). One fully developed leaf was taken from each individual and 1 cm^2^ was cut from its middle part. These small parts of leaves were put into Petri dishes with the bleaching solution (96% denatured ethanol and acetic acid, volume ratio 3:1) where they were left for 24 hours. The leaves were then transferred into solution of lactoglycerol (lactic acid, glycerol and water, volume ratio 1:1:1) where they were kept until the measurement (1 month). Stomatal length was measured on the abaxial surface of leaf on 10 stomata per leaf. The measurements were made using a light microscope (Olympus BX60, 200x magnification) interfaced with an Olympus DP70 digital camera and program QuickPhoto micro version 3.0. We present this trait as its knowledge is useful for interpreting the between cytotype differences in the control conditions, even though we miss information on its values in the other treatments.

### Data analysis

The number of flower heads and the number of flowering stalks was estimated in two different years. Because our species is a long-lived perennial, we summed the data from the two years into one number for each experimental plant. In this way, we obtained information on cumulative fitness of the plants and thus a more robust fitness estimate that we would get from a single year only. Preliminary analyses with data from the two years separately showed qualitatively the same results (not shown). We used number of flower heads rather than seed production and seed set as a measure of fitness. This is because the plants were cultivated in an experimental garden with the two cytotypes fully intermixed within each treatment and the shaded plants were covered with a net. We thus assume that the seed production would more likely reflect the cross-compatibility between the cytotypes, total number of flower heads of the given cytotype flowering in each specific moment, the availability of pollinators in the garden at the specific moment and the willingness of the pollinators to enter the shading nets rather than the response of cytotypes to the treatments.

We analyzed the effect of treatment, ploidy level and population nested within ploidy level and their interactions as fixed factor and maternal plant as a random factor for most variables. For PI_ABS,_ PI_TOTAL,_ contents of photosynthetic pigments and SLM, we did not record the population information due to technical failure, so only the effect of treatment and ploidy could be tested. For stomatal length, we only tested the effect of ploidy level and population nested within ploidy level as we used only the plants from control treatment to measure stomata length. Because of the missing population and maternal plant codes for some of the dependent variables, we compared tests with and without population as fixed factor and maternal plant as random factor where possible. The tests provided largely similar results and thus only the former tests are presented. For easy visualization, the population effects are, however, not shown in the final table. The complete table including the population code is, however, provided in the S6 Table.

The tests were done using Generalized mixed-effect linear models (GLMER) with Poisson distribution (the number of stalks) and log link function, quasi-Poisson distribution (the number of flower heads) and log link function and Gaussian distribution (Qy) with maternal plant as random effect. All the other variables, as we did not have the maternal plant code and they followed Gaussian distribution, were tested using Generalized linear models (GLM) with a Gaussian distribution. Tukey multiple comparison tests were performed post-hoc to differentiate among different levels of the predictors. All the tests were performed using R [66].

In this study, we performed each test independently for 11 different traits. According to some, we should apply the Bonferroni correction and reduce the conventional p-level from 0.05 to 0.0046 [67]. Here we used sequential Bonferroni correction (Holm-Bonferroni correction, [68]) as a less conservative modification of this approach. While even this is considered as too conservative by some authors (e.g., [69]), we report and illustrate results both with and without this correction as we have already done in our previous studies (e.g., [70]).

To easily compare plasticity of the two cytotypes in response to the two stresses between traits, we calculated the phenotypic plasticity index as the difference between the maximum and minimum value of the trait for each cytotype (separately comparing shaded and drought stressed plants to control) divided by the maximum value. This approach has been previously shown as a useful approach to compare different groups of organisms and traits (e.g., [70, 71]). All the data are available in S2–S5 Tables.

## Results

### Photosynthetic parameters

**Fig 2:**
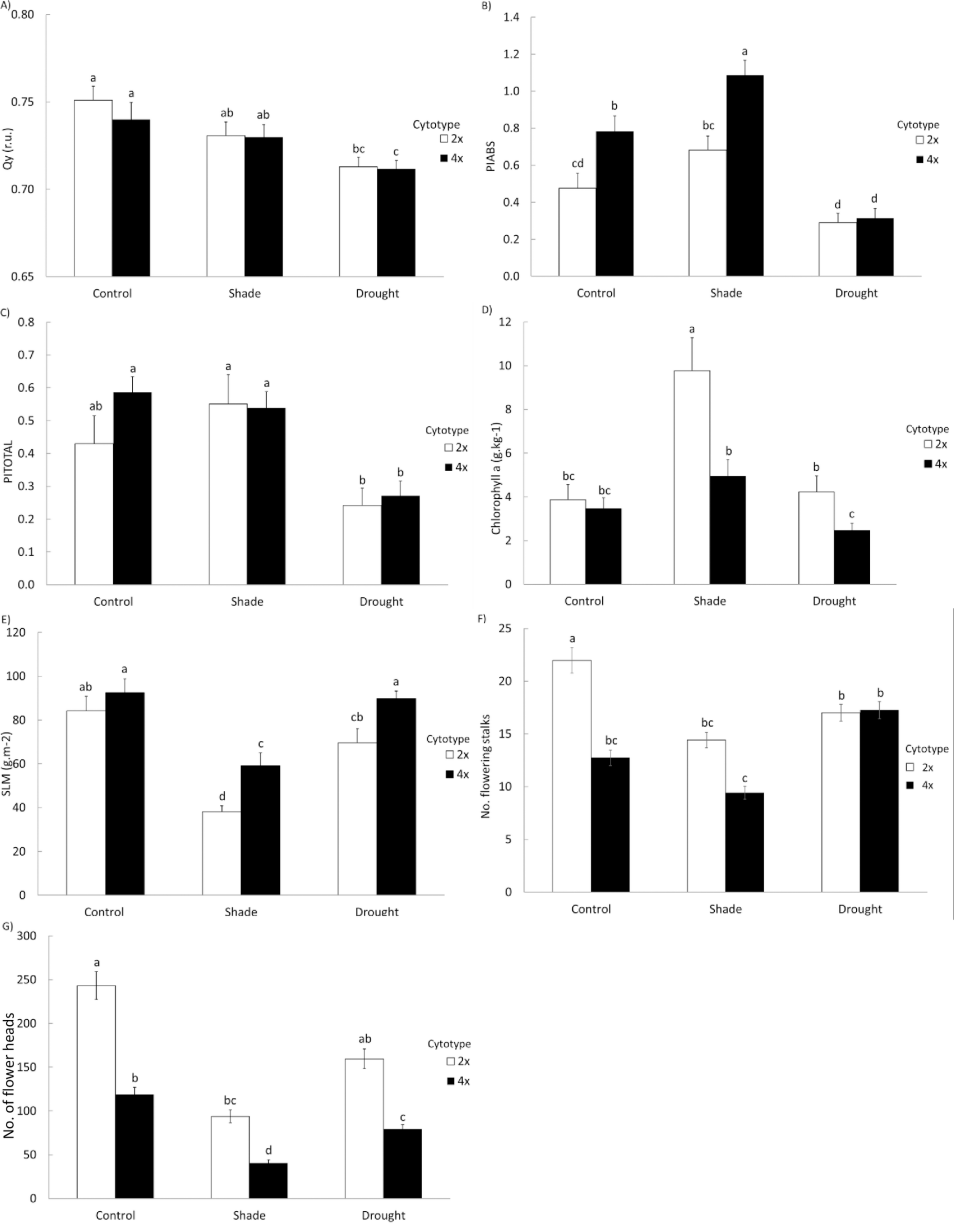
Summary of differences between cytotypes, treatments and traits. Differences between diploid (2x) and tetraploid (4x) plants growing in different treatments (shade, drought and control) in A) values of the effective quantum yield of photosystem II photochemistry in light-adapted leaves (Qy) of, B) performance index for energy conservation from Photosystem II antenna to the reduction of Q_B_ (PI_ABS_) measured in dark-adapted leaves, C) performance index for energy conservation from Photosystem II antenna to the reduction of Photosystem I end electron acceptors (PI_TOTAL_) measured in dark-adapted leaves, D) chlorophyll a content, E) specific leaf mass (SLM), F) cumulative number of flowering stalks and G) cumulative number of flower heads. The graphs show means and standard errors of the mean (SE). Columns sharing the same letter are not significantly different (P > 0.05).

Ploidy level did not have any significant effects on the effective quantum yield of PSII photochemistry (Qy) and performance index for energy conservation from Photosystem II antenna to the reduction of Photosystem I end electron acceptors (PI_TOTAL_), but had significant effect on performance index for energy conservation from Photosystem II antenna to the reduction of plastoquinone Q_B_ (PI_ABS_). All these three parameters were significantly affected by treatment. For PI_ABS_, there was also a significant interaction between ploidy and treatment when Bonferroni correction was not applied (Table 1, Fig 2A, 2B and 2C).

**Table 1:** Summary of the effects of ploidy level and treatment on the different traits. The effect of ploidy level, treatment and their interaction on effective quantum yield of photosystem II photochemistry in light-adapted leaves (Qy), performance index for energy conservation from Photosystem II antenna to the reduction of Photosystem I end electron acceptors (PI_TOTAL_)_,_ performance index for energy conservation from Photosystem II antenna to the reduction of Q_B_ (PI_ABS_)_,_ content of chlorophylls a and b and total carotenoids, specific leaf mass (SLM), cumulative number of flower heads and flowering stalks over 2012 and 2013 and plant height in 2013 measured in diploid and tetraploid plants growing in different treatments (shade, drought and control). Significant values (P < 0.05) are shown in bold. 2x or 4x next to ploidy level indicates that diploids (2x) have significantly higher values of the respective parameter than tetraploids and the other way round. Letters next to treatment indicate which plants (C-control, S-shaded, D-drought-stressed) have significantly higher values of the respective parameter. Effect of population is only shown in S6 Table. Results marked by * are significant even after sequential Bonferroni correction.

			Ploidy		Treatment		Ploidy × Treatment
	Df Error	Df	1		2		2
Qy	88	F	1.3		**10.74**	**S,C>D**	0.33
		p	0.257		0.001*		0.64
PI_ABS_	88	F	**16.32**	**4x**	**29.65**	**S,C>D**	**3.43**
		p	<0.001*		<0.001*		**0.037**
PITOTAL	88	F	1.26		**12.1**	**S,C>D**	0.38
		p	0.265		<0.001*		0.378
Stomatal length	24	F	**199.17**	**4x**			
		p	<0.001*				
Chlorophyll a	34	F	**11.63**	**2x**	**14.11**	**S>C,D**	**3.61**
		p	< 0.001*		0.002*		**0.037**
Chlorophyll b	34	F	**11.38**	**2x**	**20.18**	**S>C,D**	**3.8**
		p	< 0.001*		0.002*		**0.032**
Carotenoids	34	F	**12.01**	**2x**	**9.09**	**S>C,D**	2.97
		p	< 0.001*		0.001*		0.64
SLM	34	F	**13.78**	**4x**	**29.35**	**S<C,D**	0.84
		p	< 0.001*		< 0.001*		0.44
No. flowering stalks	132	*χ*^*2*^	**40.19**	**2x**	**84.94**	**C,D>S**	**27.13**
		p	<0.001*		<0.001*		<0.001*
No. flower heads	132	*χ*^*2*^	**1997.23**	**2x**	**2702.33**	**C>S>D**	4.03
		p	<0.001*	** **	<0.001*		0.1245

Plants of both ploidy levels stressed by drought showed the lowest Qy, PI_ABS_ and PI_TOTAL_ indicating that drought is an important stress factor for this species. On the other hand, shade resulted in higher PI_ABS_, particularly in tetraploids, but did not affect Qy and PI_TOTAL_ (Fig 2A, 2B, and 2C).

In the pairwise comparison, tetraploids growing in drought conditions had, however, significantly lower value of Qy than plants of both ploidy levels grown in shade, but diploids growing in drought were not different from diploids and tetraploids in shade (Fig 2A). For PI_ABS_, that tetraploids had significantly higher values of PI_ABS_ compared to diploids in control and shade conditions but no difference was detected in drought (Fig 2B). When the response to shade treatment was expressed as a percentage of shade *vs*. control, there were no differences between both ploidy levels. Under drought conditions, diploids were not affected as much as tetraploids compared to their respective control plants (S1F Fig). The difference in PI_TOTAL_ between the drought-stressed plants and the control ones was statistically significant only in tetraploids (Fig 2C, Table 1).

Concerning other parameters of the JIP test, tetraploids subjected to drought treatment showed an increased apparent antenna size of an active Photosystem II compared to their control plants (this can be seen both from their relative values of ABS/RC parameter and from high positive K-band visible on graph representing the difference kinetics ΔW_OJ_, S1B Fig and S1F Fig).

Another feature of tetraploid plants under drought conditions was a poor energetic connectivity between individual Photosystem II units as indicated by highly positive L-band visible on graph representing the difference kinetics ΔW_OK_ (S1C Fig). Drought-stressed plants (both diploids and tetraploids) were also characterized by rather diminished size of the pool of the end electron acceptors (i.e. acceptors after Photosystem I) compared to control plants. This can be inferred from the lower position of the W_OI_ curves between 30 and 300 ms (S1D Fig). On the other hand, diploid plants grown in shade conditions showed greater size of this pool compared to controls but this was not true for the tetraploid shade-grown plants which resembled drought-stressed plants in this respect (S1D Fig).

Content of chlorophyll *a* and *b* as well as of carotenoids was significantly higher in diploids than in tetraploids. They were also significantly higher in shade-grown plants than in plants subjected to the other two treatments. Without Bonferroni correction, there was also a significant interaction between cytotype and treatment for chlorophyll a and b (Table 1). All the patterns were largely driven by the fact that the values of chlorophyll and carotenoid contents were much higher in diploid shaded plants than in the other plants (Fig 2D).

### Morphological parameters

Both treatment and ploidy level significantly affected plant height as well as specific leaf mass (Table 1, Fig 2E). Diploids were generally taller than tetraploids, control plants were the tallest and plants under the drought stress were the shortest (Table 1). Specific leaf mass was significantly higher in tetraploids subjected to drought or shade treatments compared to diploids. Plants grown in shade conditions generally showed lower values of this parameter compared to either control or drought-stressed plants, irrespective of the ploidy level. There was no interaction between ploidy level and treatment in this case (Fig 2E, Table 1).

**Fig 3:**
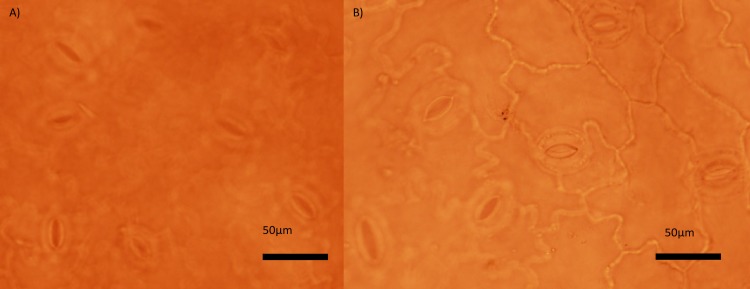
Photos of stomata. Stomata of A) diploid and B) tetraploid individual of *K*. *arvensis*.

The stomata of tetraploids were significantly larger (38.3±0.41 μm) than stomata of diploids (31.5±0.35 μm, mean±SE, Table 1, Fig 3).

### Fitness parameters

Ploidy level and treatment had significant effect on cumulative number of flowering stalks and flower heads (Table 1). Diploids produced more flower heads and flowering stalks than tetraploids over the two years (Table 1). Shaded plants produced fewer flowering stalks than the plants from the control and drought treatments (Fig 2F). The number of flower heads decreased in order control—drought—shade (Fig 2G). Number of flowering stalks also showed significant interaction between treatment and ploidy level. Specifically, diploids produced significantly more flowering stalks than tetraploids in the control treatment, while the differences between the two cytotypes were not significant in the other treatments (Table 1, Fig 2G).

### Overall plasticity

**Fig 4:**
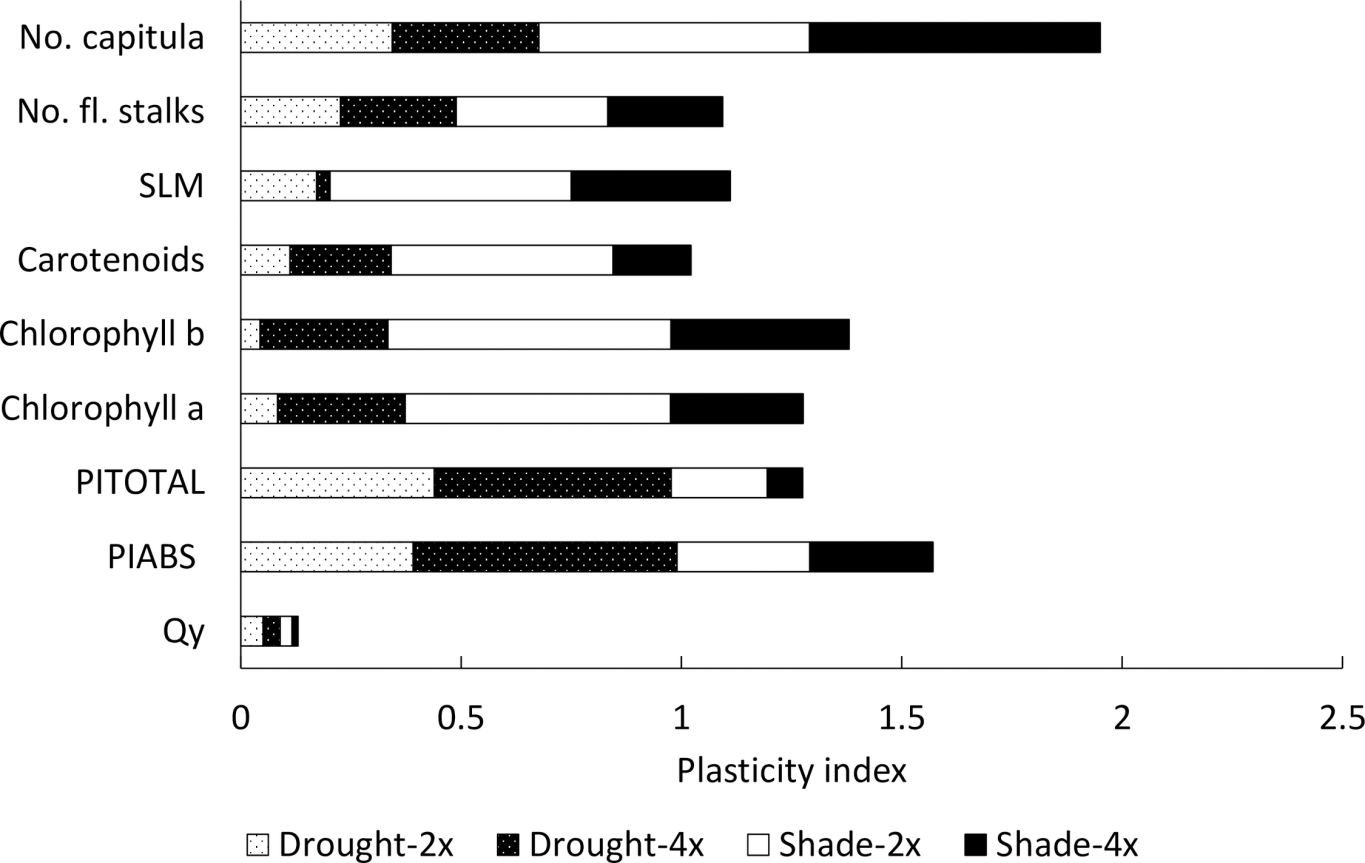
Comparison of plasticity index between cytotypes, treatments and traits. Plasticity index values (represented by the length of the column for each cytotype and treatment), comparing drought-stressed and shaded plants to control for each cytotype and trait. Plasticity indices for each cytotype and treatment are arranged next to each other to allow easy comparison among the traits.

Number of flower heads was the most plastic trait and Qy the least plastic trait. Overall, the highest plasticity was found in diploids in response to shade, while the lowest plasticity was found in diploids in response to drought (Fig 4).

## Discussion

The results of our study demonstrated that the two ploidy levels examined here significantly differed in most measured characteristics across the treatments (Table 1). Tetraploids had higher specific leaf mass (Fig 2E) and larger stomata (Fig 3). Larger stomatal size is a feature observed in many polyploid plant species including natural as well as synthetic polyploids (e.g. [72–79]. Higher specific leaf mass could be associated with another common feature of polyploids, i.e. altered regulation of cell proliferation and cell expansion [78], though the pattern is far from general [80].

Our tetraploid *K*. *arvensis* individuals were also characterized by higher PI_ABS_ values under control or shade treatments but not under drought treatment compared to diploids (Fig 2B). This parameter characterizes the overall efficiency of energy conservation in photosystem II from its antenna to the reduction of plastoquinone Q_B_ electron acceptor. Differences in the efficiency of photosynthetic electron transport between diploid and tetraploid plants grown in non-stressed conditions documented in the literature are usually small and non-significant (e.g. [20, 37, 38, 40, 81]. However, Majdi et al. [76] demonstrated significantly lower values of maximum photochemical efficiency of photosystem II in tetraploid *Tanacetum parthenium* compared to diploid individuals. Abdoli et al. [82] found similar trend for *Echinacea purpurea*, whereas Allario et al. [83] described reversed situation for *Citrus limonia* diploids and tetraploids. Clearly, the dependence of the primary photosynthetic processes on the ploidy level can differ between species. Our study thus brings further data on this topic and extends it by demonstrating that the differences between cytotypes are affected by stress. In addition, it offers the possibility to dissect photosynthetic electron transport in more detail due to the use of OJIP analysis. This, to our knowledge, has not been previously done in studies comparing different cytotypes.

The possibilities offered by OJIP analysis proved useful particularly when examining plant response to drought stress (S1 Text). Similarly to our results, Guo et al. [40] found that the exposure of *Chamerion angustifolium* plants to drought stress resulted in a greater decrease of the maximum quantum yield of Photosystem II (as well as the maximum photosynthetic rate) in leaves of hexaploid cytotype compared to the diploid one, particularly under severe drought conditions.

Despite a general expectation that polyploids perform better and have higher fitness than diploids (e.g., [26, 29–32, 84, 85]), all our fitness traits indicate higher performance of diploids (Fig 2F and 2G). Such a result has already been demonstrated previously (e.g., [86–88]). Other studies found no difference (e.g., [13, 89]).

A possible explanation for the discrepancies in the different studies and different traits may be the fact that most of the studies on diploid-polyploid species pairs was done on cytotypes occurring in close proximity, but forming secondary contact zone (e.g., [15, 90]). The performance of the cytotypes may thus be driven primarily by their different evolutionary history and not by the cytotype effects per se [80]. This may also be the case for our system as also our contact zone is of secondary origin [53]. It should also be noted that we studied only three populations and the patterns were partly population specific. Due to too low number of populations and replicates within populations, we were, however, not able to test whether the differences between populations were related to conditions of their origin. As demonstrated by [80], the effects of polyploidization may strongly differ between different genetic lineages in case of multiple polyploid origin. Future studies should thus include more populations with higher number of replicates to asses the generality of the patterns observed.

The data on plant performance, in line with the physiological data, further suggested that both drought and partly also shade represent important stress factors for the species, with its intensity differing between the different fitness traits (Fig 2F and 2G). Number of flowering stalks indicated that the response to treatments differed between cytotypes while the interaction between treatment and cytotype was not significant for number of flower heads. Still, reduction of both number of flowering stalks and flower heads under stress was stronger in diploids in than tetraploids. However, as the diploids showed higher values in control conditions, they did not differ under stress or diploids were still larger (Fig 2F and 2G). This thus suggests that the diploids show higher fitness and thus could outperform tetraploids in favorable conditions but the two cytotypes may co-exist under stress. This is in line with our unpublished observation that tetraploids are restricted to drier more shaded sites, while diploids occupy much wider spectrum of habitats. Similar fit between performance in controlled conditions and wider distribution of diploids in the field was also found by several other studies (e.g., [57, 91, 92]).

Comparison of trait plasticities between cytotypes and treatments suggested that the highest overall plasticity can be detected in diploids in response to shading (Fig 4). This contradicts the expectation that polyploids should show higher plasticity due to increased heterozygosity levels (e.g. [16]). It may be explained by the observations of Sultan [93, 94], who demonstrated that higher plasticity in some traits they studied may in fact allow stability in some other studied traits. This explanation is supported by the fact that higher plasticity in diploids is mostly apparent in long-term traits related to fitness and content of photosynthetic pigments, i.e. traits with more complex regulation.

### Limitations of the study

For this experiment, we only used plants originating from 25 maternal plants for each cytotype. This number is relatively low and thus could potentially limit the power of our tests. However, our maternal plants come from multiple populations. In addition, offspring of each maternal plant are represented in each treatment. Thus, we are confident that any differences between cytotypes we detected are not due to a single outlying population or single outlying maternal plant. Similarly, any differences between treatments may not be due to different maternal plants present in each. Thus, our data may be weak and we may miss differences, that would be significant should we have larger sample size. However, any significant difference that was detected can be trusted. As the number of significant effects is quite high, we suggest that our study was powerful enough to detect differences between cytotypes and treatments. It was, however, likely too weak to detect relationships between the different traits.

While we tested 11 different short-term as well as long-term traits in our study, wide range of additional traits could be measured and explored. These could include water use efficiency as an important trait reflecting species ability to respond to drought stress as well as many additional traits such as number of chloroplasts/cell, cell size and many other. These traits could indeed bring additional interesting insights into the between cytotype differences. Measuring them was, however, out of our working capacity and the scope of the study.

## Conclusions

Overall, the study indicated that diploid individuals of *K*. *arvensis* are, in terms of fitness, more negatively affected by stress than tetraploids. The fitness of diploids under favorable conditions is, however, higher than that of tetraploids. This contrasts with the fact that tetraploids have seemingly more efficient photosynthetic apparatus under favorable conditions. When stressed especially by drought, misbalances in tetraploid photosynthesis occur leading to equal photosynthetic activity between cytotypes under drought stress. Some of the patterns are thus in line with the expectations that tetraploids are better able to cope with environmental stress than diploids, while other patterns suggest the opposite. Similarly, the photosynthesis related traits are in line with theoretical expectation on higher plasticity in polyploids, while the fitness related traits show higher plasticity in diploids especially in response to drought.

Comparison across the different traits combining short-term physiological traits with long-term growth and fitness related traits suggests that the inferences on stress responses drawn based on one trait are often contradictory to inferences based on another trait. In addition, we did not confirm our expectation that short-term traits, more directly related to direct cell changes, will show more predictable responses than the long-term growth and fitness related traits. This has two important implications. First, it suggests that focusing on a small number of traits or on single environmental conditions does not allow making general inferences on the function of different cytotypes, as the responses are strongly trait and condition specific. At the same time, the results also suggest that when measuring multiple traits, it may in fact be very difficult to interpret the results and to arrive at a general conclusion. While we attempted to pick up traits that are mechanically linked to each other, translating traits from physiological level to real growth patterns is not an easy task (e.g. [95, 40]). In fact, we even failed to find significant relationships between the traits within a selection analysis (not shown). Further studies attempting to understand the specific mechanisms behind the relationships between different plant traits are thus necessary to increase our ability to make inferences on species performance under different conditions.

## Supporting information

S1 Fig Detailed results of JIP analyses. The polyphasic rise of chlorophyll *a* fluorescence transients (OJIP) **[A]**, the difference kinetics ΔW_OJ_ **[B]** and ΔW_OK_ **[C]** revealing the K- and L- bands, respectively, the relative variable fluorescence W_OI_ **[D]**, and W_IP_ **[E]** and the relative changes (expressed as the percentage of stress/control) of the selected parameters of the JIP test **[F]**. Chlorophyll fluorescence was measured in dark-adapted leaves of diploid (2x) and tetraploid (4x) plants growing in different treatments (shade, drought and control). For the explanation of the individual parameters of the JIP test see [63]. r.u. relative units.
(DOCX)

S1 Table List of measured photosynthetic parameters. Selected photosynthetic parameters of the JIP test derived from the measurements of the polyphasic rise of chlorophyll *a* fluorescence transient based on the theory described in Strasser et al., 2000, and Stirbet and Govindjee, 2011. F_0_—the initial fluorescence intensity (at 40 μs), F_K_—the fluorescence intensity at the K-step (300 μs), F_J_—the fluorescence intensity at the J-step (at 2 ms), F_I_—the fluorescence intensity at the I-step (at 30 ms), F_M_ ≈ F_P_—the maximum fluorescence intensity, Area—area between the fluorescence curve and F_M_, PSI—photosystem I, PSII—photosystem II, RC—reaction centre.
(DOCX)

S2 Table Primary data on Qy, number of flowering stalks and number of flower heads. (PDF)

S3 Table Primary data on Pi_TOTAL_ and Pi_ABS_. (PDF)

S4 Table Primary data on specific leaf mass, chlorophyll and carotenoid content. (PDF)

S5 Table Primary data on stomata length. (PDF)

S6 Table Summary of the effects of ploidy level, treatment and population on the different traits. The effect of ploidy level, population, treatment and their interaction on effective quantum yield of photosystem II photochemistry in light-adapted leaves (Qy), performance index for energy conservation from Photosystem II antenna to the reduction of Photosystem I end electron acceptors (PI_TOTAL_)_,_ performance index for energy conservation from Photosystem II antenna to the reduction of Q_B_ (PI_ABS_)_,_ content of chlorophylls a and b and total carotenoids, specific leaf mass (SLM), cumulative number of flower heads and flowering stalks over 2012 and 2013 and plant height in 2013 measured in diploid and tetraploid plants growing in different treatments (shade, drought and control). Significant values (P < 0.05) are shown in bold. 2x or 4x next to ploidy level indicates that diploids (2x) have significantly higher values of the respective parameter than tetraploids and the other way round. Letters next to treatment indicate which plants (C-control, S-shaded, D-drought-stressed) have significantly higher values of the respective parameter. Results marked by * are significant even after sequential Bonferroni correction.
(DOCX)

S1 Text Discussion of results on photosynthetic activity. Detailed discussion of the results on the different measures of photosynthetic activity.
(DOCX)
