# Supplementary material for: Physiological and fitness differences between cytotypes vary with stress in a grassland perennial herb
Source: PLoS One. 2017 Nov 30;12(11):e0188795. doi: 10.1371/journal.pone.0188795 (PMC5708818; doi:10.1371/journal.pone.0188795)

Supplementary Figure S1. The polyphasic rise of chlorophyll *a* fluorescence transients (OJIP) **[A]**, the difference kinetics ΔW_OJ_  **[B]** and ΔW_OK_ **[C]** revealing the K- and L- bands, respectively, the relative variable fluorescence W_OI_ **[D]**, and W_IP_ **[E]** and the relative changes (expressed as the percentage of stress/control) of the selected parameters of the JIP test **[F]**. Chlorophyll fluorescence was measured in dark-adapted leaves of diploid (2x) and tetraploid (4x) plants growing in different treatments (shade, drought and control). For the explanation of the individual parameters of the JIP test see (Stirbet and Govindjee, 2011). r.u. relative units.


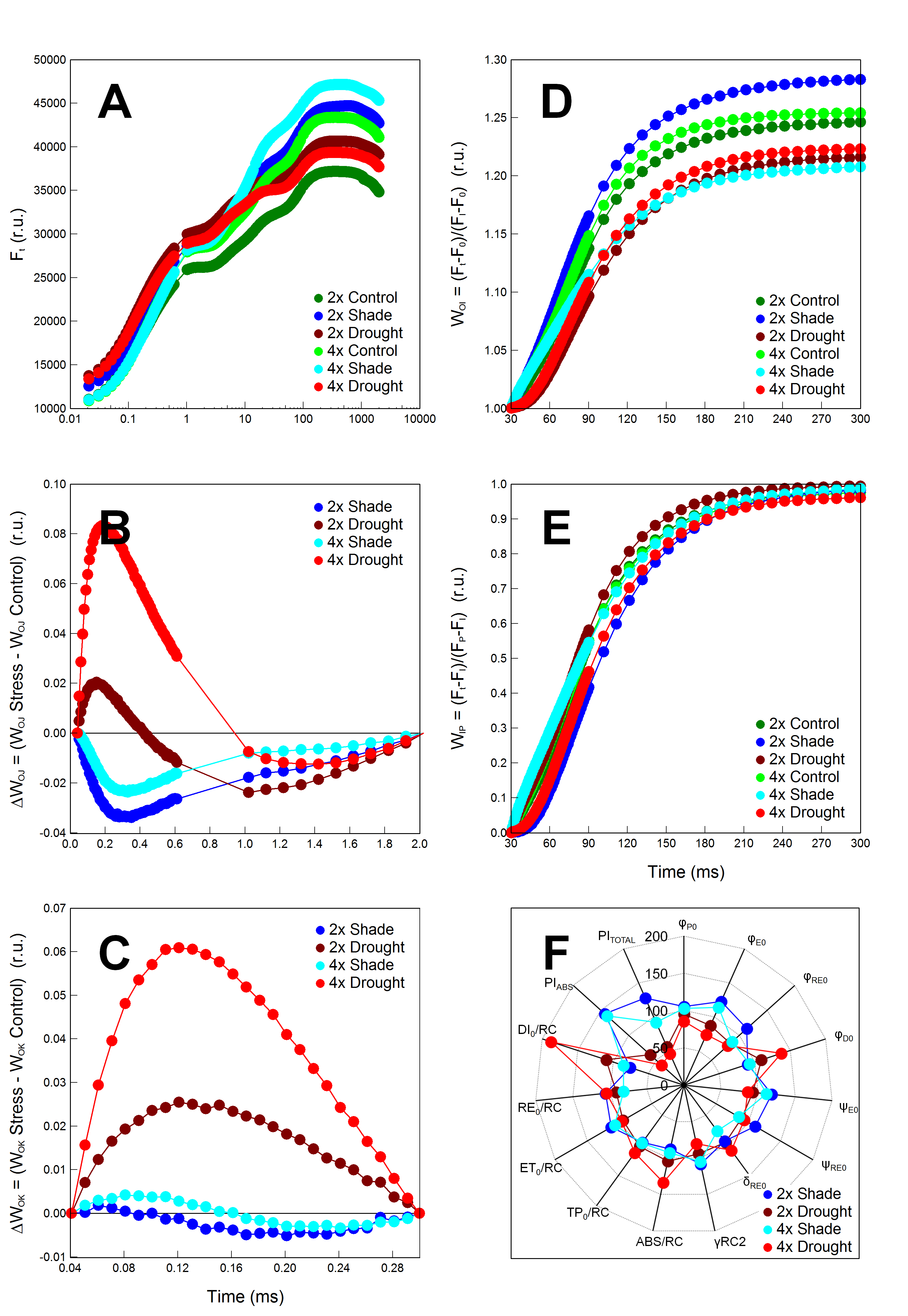

Supplement: S1 Fig — The polyphasic rise of chlorophyll a fluorescence transients (OJIP) [A], the difference kinetics ΔWOJ [B] and ΔWOK [C] revealing the K- and L- bands, respectively, the relative variable fluorescence WOI [D], and WIP [E] and the relative changes (expressed as the percentage of stress/control) of the selected parameters of the JIP test [F]. Chlorophyll fluorescence was measured in dark-adapted leaves of diploid (2x) and tetraploid (4x) plants growing in different treatments (shade, drought and control). For the explanation of the individual parameters of the JIP test see [63]. r.u. relative units. (DOCX) [file pone.0188795.s001.docx]
