# Supplementary material for: Physiological and fitness differences between cytotypes vary with stress in a grassland perennial herb
Source: PLoS One. 2017 Nov 30;12(11):e0188795. doi: 10.1371/journal.pone.0188795 (PMC5708818; doi:10.1371/journal.pone.0188795)
Supplement: S2 Table — (PDF) [file pone.0188795.s003.pdf]

Ploidy	Treatment	Qy	Stalk	Flower
D	shade	0.61	10	51
D	shade	0.62	16	44
D	control	0.645	23	224
T	control	0.645	12	98
D	drought	0.65	14	164
T	shade	0.665	8	18
T	shade	0.67	7	37
T	shade	0.675	10	40
T	drought	0.675	24	84
T	shade	0.685	6	14
T	control	0.69	9	105
T	shade	0.69	24	50
D	drought	0.69	13	225
T	drought	0.69	10	40
D	drought	0.695	23	290
T	drought	0.695	7	27
T	drought	0.7	7	21
T	drought	0.7	13	46
T	drought	0.7	15	48
D	drought	0.705	16	43
D	drought	0.705	11	60
D	drought	0.705	13	284
D	control	0.71	14	97
D	drought	0.71	23	308
T	drought	0.71	18	88
T	control	0.715	14	276
D	drought	0.715	29	418
T	drought	0.715	10	41
T	control	0.72	8	30
T	control	0.72	39	228
D	shade	0.72	14	102
T	shade	0.72	2	7
D	drought	0.72	22	120
T	drought	0.72	13	73
T	drought	0.72	17	75
T	drought	0.72	22	97
T	drought	0.72	36	144
D	control	0.725	29	449
D	drought	0.725	15	38
D	drought	0.725	15	151
D	drought	0.725	28	230
D	shade	0.73	12	93
T	shade	0.73	5	14
T	shade	0.73	6	23
D	drought	0.73	7	35
D	drought	0.73	18	83
T	drought	0.73	24	63
T	drought	0.73	16	76
D	control	0.735	16	57

Ploidy	Treatment	Qy	Stalk	Flower
D	control	0.735	23	131
D	shade	0.735	13	168
T	shade	0.735	5	19
T	shade	0.735	10	42
D	drought	0.735	6	16
T	drought	0.735	28	173
T	control	0.74	20	288
D	shade	0.74	23	154
T	shade	0.74	5	21
T	shade	0.74	23	96
T	shade	0.74	24	177
D	drought	0.74	34	368
D	control	0.745	16	89
D	shade	0.745	11	37
D	shade	0.745	19	51
T	drought	0.745	4	23
T	drought	0.745	19	138
D	control	0.75	20	282
T	control	0.75	12	107
T	control	0.75	8	119
D	shade	0.75	9	19
D	shade	0.75	16	78
D	control	0.755	5	97
D	control	0.755	16	174
T	control	0.755	2	13
T	control	0.755	11	48
D	shade	0.755	20	140
D	shade	0.755	24	312
D	control	0.76	14	214
D	shade	0.76	6	61
T	shade	0.76	3	3
D	control	0.765	15	466
D	control	0.765	40	752
D	shade	0.765	31	188
T	control	0.77	14	100
T	shade	0.77	7	29
T	shade	0.77	4	34
D	control	0.775	15	270
T	control	0.775	15	120
T	shade	0.775	2	4
D	control	0.78	66	306
T	control	0.78	16	170
T	shade	0.79	15	52
T	control	0.8	24	232
D	control	0.81	27	225
