# Supplementary material for: Physiological and fitness differences between cytotypes vary with stress in a grassland perennial herb
Source: PLoS One. 2017 Nov 30;12(11):e0188795. doi: 10.1371/journal.pone.0188795 (PMC5708818; doi:10.1371/journal.pone.0188795)
Supplement: S3 Table — (PDF) [file pone.0188795.s004.pdf]

Ploidy	Treatment	PITOT/	PIABS
D	Control	0.13	0.16
D	Control	0.23	0.15
D	Control	1.14	0.81
D	Control	0.18	0.27
D	Control	0.22	0.46
D	Control	0.28	0.49
D	Control	0.10	0.12
D	Control	0.39	0.36
D	Control	0.52	0.68
D	Control	0.75	1
D	Control	0.65	0.72
D	Control	0.95	1.03
D	Control	0.11	0.08
D	Control	0.10	0.1
D	Control	0.28	0.46
D	Control	0.28	0.3
D	Control	0.58	0.66
D	shade	0.52	0.65
D	shade	0.36	0.85
D	shade	0.45	0.45
D	shade	0.56	0.82
D	shade	0.14	0.57
D	shade	0.31	0.82
D	shade	0.32	0.48
D	shade	0.52	0.81
D	shade	1.34	0.84
D	shade	0.58	0.28
D	shade	0.66	1.23
D	shade	0.85	0.41
D	drought	0.13	0.38
D	drought	0.18	0.19
D	drought	0.73	0.7
D	drought	0.2	0.14
D	drought	0.14	0.26
D	drought	0.2	0.26
D	drought	0.22	0.13
D	drought	0.48	0.56
D	drought	0.16	0.27
D	drought	0.15	0.21
D	drought	0.1	0.15
D	drought	0.19	0.24
T	Control	0.88	1.11
T	Control	0.88	0.76
T	Control	0.56	0.9

T	Control	0.69	1.05
T	Control	0.65	0.72
T	Control	0.58	0.69
T	Control	0.62	0.68
T	Control	0.72	1.57
T	Control	0.49	0.41
T	Control	0.61	0.98
T	Control	0.47	0.6
T	Control	0.47	0.52
T	Control	0.13	0.21
T	Control	0.67	0.87
T	Control	0.28	0.42
T	shade	0.66	0.66
T	shade	0.72	1.89
T	shade	0.62	1.08
T	shade	0.36	1.04
T	shade	0.37	0.79
T	shade	0.61	0.91
T	shade	0.46	0.95
T	shade	0.64	1.23
T	shade	0.41	1.19
T	shade	0.34	1.12
T	shade	0.46	0.87
T	shade	1.12	1.89
T	shade	0.27	0.85
T	shade	0.33	1.08
T	shade	0.57	1.26
T	shade	0.5	0.76
T	shade	0.45	0.85
T	shade	0.8	1.17
T	drought	0.15	0.25
T	drought	0.15	0.18
T	drought	0.33	0.35
T	drought	0.06	0.15
T	drought	0.22	0.18
T	drought	0.17	0.44
T	drought	0.18	0.16
T	drought	0.14	0.05
T	drought	0.26	0.39
T	drought	0.1	0.11
T	drought	0.27	0.35
T	drought	0.95	1.1
T	drought	0.3	0.56
T	drought	0.52	0.33
T	drought	0.29	0.42

T	drought	0.22	0.19
T	drought	0.35	0.24
T	drought	0.2	0.28
