# Supplementary material for: Physiological and fitness differences between cytotypes vary with stress in a grassland perennial herb
Source: PLoS One. 2017 Nov 30;12(11):e0188795. doi: 10.1371/journal.pone.0188795 (PMC5708818; doi:10.1371/journal.pone.0188795)
Supplement: S4 Table — (PDF) [file pone.0188795.s005.pdf]

Ploidy	Treatment	SLM	ChlA	ChlB	Car
Diploid	Control	110.0717	1.388653	0.412505	0.485363
Diploid	Control	94.84475	1.752324	0.615914	0.566949
Diploid	Control	77.69374	4.114695	1.316073	0.945674
Diploid	Control	68.27229	5.36435	1.76133	1.254905
Diploid	Control	81.87367	5.739703	1.869859	1.3071
Diploid	Control	96.07219	3.373659	1.076704	0.946941
Diploid	Control	60.04512	5.419213	1.755559	1.152424
Diploid	drought	46.17834	6.356285	1.968539	1.480521
Diploid	drought	83.00159	2.036379	0.610434	0.65307
Diploid	drought	92.22399	1.856799	0.56174	0.563843
Diploid	drought	68.20594	2.917971	0.918059	0.766521
Diploid	drought	83.39968	4.69343	1.434614	1.080081
Diploid	drought	59.58068	5.597779	1.753308	1.384945
Diploid	drought	54.80361	6.162495	1.96675	1.56684
Diploid	shade	38.81369	10.90739	3.949288	2.042239
Diploid	shade	38.88004	6.824489	2.496044	1.37076
Diploid	shade	52.34873	4.39601	1.657421	0.987917
Diploid	shade	31.91348	14.13841	4.68891	2.882645
Diploid	shade	40.53875	6.10405	2.334175	1.17758
Diploid	shade	32.64331	14.58924	5.452235	2.727848
Diploid	shade	31.1173	11.37847	4.057135	2.235538
Tetraploid	Control	107.4177	1.86773	0.663378	0.545931
Tetraploid	Control	96.13854	4.639673	1.446939	1.055082
Tetraploid	Control	106.5552	2.080664	0.674663	0.56935
Tetraploid	Control	92.62208	3.44164	1.003933	0.88719
Tetraploid	Control	100.2521	3.308082	1.008008	0.814134
Tetraploid	Control	85.58917	3.329896	1.107409	0.852993
Tetraploid	Control	59.04989	5.560001	1.806404	1.30169
Tetraploid	drought	107.2187	1.806634	0.598973	0.54465
Tetraploid	drought	92.35669	2.445439	0.809362	0.682372
Tetraploid	drought	87.97771	1.973482	0.665037	0.548907
Tetraploid	drought	80.48036	1.308084	0.352284	0.495528
Tetraploid	drought	91.36146	3.461718	1.032883	0.811657
Tetraploid	drought	80.61306	2.533148	0.806231	0.699636
Tetraploid	drought	88.2431	3.68828	1.210272	0.861192
Tetraploid	shade	58.45276	6.562385	2.497823	1.27208
Tetraploid	shade	49.5621	5.245783	2.018209	1.136006
Tetraploid	shade	45.71391	6.749143	2.394894	1.391809
Tetraploid	shade	38.41561	7.145448	2.717191	1.558265
Tetraploid	shade	76.49947	2.889268	1.045595	0.645934
Tetraploid	shade	79.55149	3.804157	1.373651	0.810869
Tetraploid	shade	65.75106	2.239542	0.886365	0.504351
