# Supplementary material for: Physiological and fitness differences between cytotypes vary with stress in a grassland perennial herb
Source: PLoS One. 2017 Nov 30;12(11):e0188795. doi: 10.1371/journal.pone.0188795 (PMC5708818; doi:10.1371/journal.pone.0188795)
Supplement: S5 Table — (PDF) [file pone.0188795.s006.pdf]

Ploidy	Stomata length
Diploid	32.2
Diploid	27.3
Diploid	31.2
Diploid	28.8
Diploid	38.7
Diploid	31
Diploid	36.9
Diploid	29.5
Diploid	31.5
Diploid	31.3
Diploid	32.2
Diploid	28.3
Diploid	32.5
Diploid	28.9
Diploid	32.1
Tetraploid	44.1
Tetraploid	38.9
Tetraploid	40.5
Tetraploid	40.2
Tetraploid	38.4
Tetraploid	29.9
Tetraploid	35.4
Tetraploid	39.9
Tetraploid	36.5
Tetraploid	34.4
Tetraploid	33.3
Tetraploid	42.1
Tetraploid	44.1
Tetraploid	41.3
Tetraploid	35.7
