# Supplementary material for: Physiological and fitness differences between cytotypes vary with stress in a grassland perennial herb
Source: PLoS One. 2017 Nov 30;12(11):e0188795. doi: 10.1371/journal.pone.0188795 (PMC5708818; doi:10.1371/journal.pone.0188795)
Supplement: S6 Table — The effect of ploidy level, population, treatment and their interaction on effective quantum yield of photosystem II photochemistry in light-adapted leaves (Qy), performance index for energy conservation from Photosystem II antenna to the reduction of Photosystem I end electron acceptors (PITOTAL), performance index for energy conservation from Photosystem II antenna to the reduction of QB (PIABS), content of chlorophylls a and b and total carotenoids, specific leaf mass (SLM), cumulative number of flower heads and flowering stalks over 2012 and 2013 and plant height in 2013 measured in diploid and tetraploid plants growing in different treatments (shade, drought and control). Significant values (P < 0.05) are shown in bold. 2x or 4x next to ploidy level indicates that diploids (2x) have significantly higher values of the respective parameter than tetraploids and the other way round. Letters next to treatment indicate which plants (C-control, S-shaded, D-drought-stressed) have significantly higher values of the respective parameter. Results marked by * are significant even after sequential Bonferroni correction. (DOCX) [file pone.0188795.s007.docx]

Supplementary Table S6. The effect of ploidy level, population, treatment and their interaction on effective quantum yield of photosystem II photochemistry in light-adapted leaves (Qy), performance index for energy conservation from Photosystem II antenna to the reduction of Photosystem I end electron acceptors (PI_TOTAL_)_,_ performance index for energy conservation from Photosystem II antenna to the reduction of Q_B_ (PI_ABS_)_,_ content of chlorophylls a and b and total carotenoids, specific leaf mass (SLM), cumulative number of capitula and flowering stalks over 2012 and 2013 and plant height in 2013 measured in diploid and tetraploid plants growing in different treatments (shade, drought and control). Significant values (P < 0.05) are shown in bold. 2x or 4x next to ploidy level indicates that diploids (2x) have significantly higher values of the respective parameter than tetraploids and the other way round. Letters next to treatment indicate which plants (C-control, S-shaded, D-drought-stressed) have significantly higher values of the respective parameter. Results marked by * are significant even after sequential Bonferroni correction.

|  |  |  | Ploidy | | Population | Treatment | | Ploidy × Treatment | Pop. × Treatment |
| --- | --- | --- | --- | --- | --- | --- | --- | --- | --- |
|  | Df Error | Df | 1 |  | 8 | 2 |  | 2 | 16 |
| Qy | 88 | F | 1.3 |  | 1.52 | **10.74** | **S,C>D** | 0.33 | 0.62 |
|  |  | p | 0.257 |  | 0.084 | **0.001*** |  | 0.64 | 0.841 |
| PI_ABS_ | 88 | F | **16.32** | **4x** |  | **29.65** | **S,C>D** | **3.43** |  |
|  |  | p | **<0.001*** |  |  | **<0.001*** |  | **0.037** |  |
| PITOTAL | 88 | F | 1.26 |  |  | **12.1** | **S,C>D** | 0.38 |  |
|  |  | p | 0.265 |  |  | **<0.001*** |  | 0.378 |  |
| Stomatal length | 24 | F | **199.17** | **4x** | **16.48** |  |  |  |  |
|  |  | p | **<0.001*** |  | **<0.001** |  |  |  |  |
| Chlorophyll a | 34 | F | **11.63** | **2x** |  | **14.11** | **S>C,D** | **3.61** |  |
|  |  | p | **< 0.001*** |  |  | **0.002*** |  | **0.037** |  |
| Chlorophyll b | 34 | F | **11.38** | **2x** |  | **20.18** | **S>C,D** | **3.8** |  |
|  |  | p | **< 0.001*** |  |  | **0.002*** |  | **0.032** |  |
| Carotenoids | 34 | F | **12.01** | **2x** |  | **9.09** | **S>C,D** | 2.97 |  |
|  |  | p | **< 0.001*** |  |  | **0.001*** |  | 0.64 |  |
| SLM | 34 | F | **13.78** | **4x** |  | **29.35** | **S<C,D** | 0.84 |  |
|  |  | p | **< 0.001*** |  |  | **< 0.001*** |  | 0.44 |  |
| No. flowering stalks | 132 | *χ^2^* | **40.19** | **2x** | 54.26 | **84.94** | **C,D>S** | **27.13** | **29.18** |
|  |  | p | **<0.001*** |  | **<0.001*** | **<0.001*** |  | **<0.001*** | **0.02** |
| No. flower heads | 132 | *χ^2^* | **1997.23** | **2x** | 42.19 | **2702.33** | **C>S>D** | 4.03 | **49.58** |
|  |  | p | **<0.001*** |  | **<0.001*** | **<0.001*** |  | 0.1245 | **<0.001*** |
