# Supplementary material for: Physiological and fitness differences between cytotypes vary with stress in a grassland perennial herb
Source: PLoS One. 2017 Nov 30;12(11):e0188795. doi: 10.1371/journal.pone.0188795 (PMC5708818; doi:10.1371/journal.pone.0188795)
Supplement: S1 Text — Detailed discussion of the results on the different measures of photosynthetic activity. (DOCX) [file pone.0188795.s008.docx]

Supplementary Text S1. Detailed discussion of the results on the different measures of photosynthetic activity.

The adaptability of different cytotypes to adverse environment could be influenced by the ability of their photosynthetic apparatus to adapt to such conditions. However, the relationship between various processes taking place during photosynthesis is a very intricate one and the fact that one cytotype shows better photosynthetic performance under favorable conditions does not necessarily indicate that it will retain such ability under stress. Indeed, well-balanced photosynthesis offering advantage to cytotypes with higher ploidy level, often described in the literature, can be disrupted by physiological changes taking place in stressed plants. This was evident in the case of our drought-stressed *Knautia* plants. Drought treatment resulted in a poorer overall performance of photosynthetic electron transport and tetraploids showed a higher decrease of the efficiency of primary photosynthetic processes. According to the results of OJIP analysis, this was associated with two main factors: the poorer excitonic connectivity between their individual photosystem II units and the increased dissipation of excess excitation energy captured by their light-harvesting antennae. These two processes are probably interconnected, as the inability of photosystem II units to transfer the excitation energy among each other would mean that the captured light energy could not be efficiently utilized and would have to be dissipated by non-photochemical processes. However, even this dissipation could be inadequate and a damage to photosystem II could then occur.

Regarding the ability to capture and dissipate the excitation energy, the apparent antenna size of an active Photosystem II complex (ABS/RC parameter) increased more in tetraploid plants subjected to drought than in diploids, but at the same time diploids had generally higher content of photosynthetic pigments. As not all chlorophylls are bound to light-harvesting antennae but a great amount of them is associated with either photosystem I or CP43 and CP47 proteins of photosystem II reaction center (or with other proteins in preparation to their insertion to main photosynthetic complexes of thylakoid membranes) ((Nelson and Yocum, 2006)), these two observations are not necessarily contradictory. Moreover, the ABS/RC parameter is only a relative one and expresses the antenna size in relation to active photosystem II reaction centers. We can speculate that although the external light-harvesting proteins in tetraploid drought-stressed plants could still function as a source of energy, there was an insufficient sink for this energy due to more inactive or even damaged photosystem II reaction centers compared to diploids. This in turn necessitated the need for a greater dissipation of the excess excitation energy as seen in these plants. However, even this was probably insufficient and resulted in the overall poorer performance of photosynthetic electron transport chain in thylakoid membranes in drought stressed tetraploids compared to diploids. Similar effect was found by (Zhang and Gao, 1999), who assessed slow phase of chlorophyll fluorescence kinetics in diploid and triploid poplar clones and found both a higher level of energy dissipation and a greater susceptibility to photoinhibition in triploid compared to diploid clones. Another study, (Zhang et al., 2010), who worked with diploid and tetraploid plants of *Lonicera japonica* stressed by ozone, also observed a higher decrease of the NPQ parameter (representing the dissipation of excess energy non-utilizable by photosynthetic electron transport that would otherwise have damaging consequences) in diploids together with a greater negative effect on the efficiency of primary photosynthetic processes in tetraploids under these stress conditions. Similarly, (Coate et al., 2013) studied various components of NPQ in allotetraploid *Glycine dolichocarpa* and its diploid progenitors and found that tetraploids do not differ from diploids in the photoinhibition-associated compound of NPQ but show higher efficiency of energy-dependent and zeaxanthin-dependent NPQ. It was also characterized by higher expression of genes associated with xanthophyll cycle (i.e., transformation of violaxanthin to zeaxanthin which can serve as an efficient protection against reactive oxygen species) as well as genes coding for proteins of water-water cycle and cyclic electron transport around photosystem I (both these cycles can serve as efficient means of protection against overreduction of photosynthetic linear electron transport chain) (Coate et al., 2013). On the other hand, (Li et al., 2009) working with drought-stressed diploid and tetraploid plants of *Lonicera japonica* observed exactly the opposite situation, i.e. an increased NPQ and a decreased efficiency of the photosynthetic electron transport in diploids, whereas under heat stress conditions, the performance of photosynthetic electron transport chain in tetraploids compared to diploids was lower but the NPQ was lower as well ((Li et al., 2011)). Thus, the relationship between the processes taking place in thylakoid membranes and the adaptability of plants with different ploidy levels to deal with unfavorable conditions is still not clear and requires further studies.

References

**Coate JE, Powell AF, Owens TG, Doyle JJ.** **2013**. Transgressive physiological and transcriptomic responses to light stress in allopolyploid Glycine dolichocarpa (Leguminosae). *Heredity,* **110**: 160-170.

**Li WAB, Biswas DK, Xu H, Xu C, Wang X, Liu J, Jiang G.** **2009**. Photosynthetic responses to chromosome doubling in relation to leaf anatomy in *Lonicera japonica* subjected to water stress. *Functional plant biology,* **36**: 1-10.

**Li WD, Hu X, Liu JK, Jiang GM, Li O, Xing D.** **2011**. Chromosome doubling can increase heat tolerance in Lonicera japonica as indicated by chlorophyll fluorescence imaging. *Biologia Plantarum,* **55**: 279-284.

**Nelson N, Yocum CF.** **2006**. Structure and function of photosystems I and II. *Annual Review of Plant Biology,* **57**: 521-565.

**Zhang L, Xu H, Yang JC, Li WD, Jiang GM, Li YG.** **2010**. Photosynthetic characteristics of diploid honeysuckle (Lonicera japonica Thunb.) and its autotetraploid cultivar subjected to elevated ozone exposure. *Photosynthetica,* **48**: 87-95.

**Zhang SR, Gao RF.** **1999**. Diurnal changes of gas exchange, chlorophyll fluorescence, and stomatal aperture of hybrid poplar clones subjected to midday light stress. *Photosynthetica,* **37**: 559-571.
